# Superoxide dismutase activity as a predictor of adverse outcomes in patients with nonischemic dilated cardiomyopathy

**DOI:** 10.1007/s12192-019-00991-3

**Published:** 2019-05-01

**Authors:** Ewa Romuk, Wojciech Jacheć, Ewa Kozielska-Nowalany, Ewa Birkner, Aleksandra Zemła-Woszek, Celina Wojciechowska

**Affiliations:** 1 Department of Biochemistry, School of Medicine with the Division of Dentistry, Medical University of Silesia, Jordana 19 Street, 41-808 Zabrze, Poland; 2 Second Department of Cardiology, School of Medicine with the Division of Dentistry, Medical University of Silesia, M. C. Skłodowskiej 10 Street, 41-808 Zabrze, Poland

**Keywords:** Dilated cardiomyopathy, Heart failure, Oxidative stress, Superoxide dismutase

## Abstract

Oxidative stress contributes to progression of heart failure (HF). The present study analyzed the efficacy of the activities of superoxide dismutase (SOD) and its isoenzymes (CuZnSOD and MnSOD) as prognostic factors in dilated cardiomyopathy. The usefulness of activities of total SOD, MnSOD, and CuZnSOD was assessed, taking into account clinical, echocardiographic, and laboratory parameters as risk predictors of long-term clinical outcomes (death, heart transplant, combined end point) in 109 patients with nonischemic dilated cardiomyopathy (NIDCM) in this study with a 5-year follow-up. Regression analysis showed that total serum SOD activity was a predictor of worse long-term clinical outcome even after adjustment for NT-proBNP, hemoglobin, sodium, creatinine clearance, left ventricular ejection fraction (LVEF), BMI, and NYHA class (LVEF: HR 1.059, 95% CI 1.007–1.114, *P* = 0.026; BMI: HR 1.073, 95% CI 1.021–1.126, *P* = 0.005; NYHA: HR 1.073, 95% CI 1.022–1.126, *P* = 0.005). MnSOD and CuZnSOD activities were also predictors of worse long-term clinical outcome even after adjustment for laboratory parameters and BMI or NYHA class; however, after adjustment for LVEF, a borderline statistical significance was achieved (LVEF: HR 1.054, 95% CI 0.993–1.119, *P* = 0.081 [MnSOD]; HR 1.092, 95% CI 0.989–1.297, *P* = 0.082 [CuZnSOD]). Increased activities of total serum SOD and its isoenzymes in NIDCM patients correspond with a poor prognosis and may have prognostic value in the prediction of long-term clinical outcomes. In conclusion, the present study shows that serum SOD activity may be a useful predictor of adverse outcome in HF.

## Introduction

Nonischemic dilated cardiomyopathy (NIDCM) is a common disease of the heart muscle (Jefferies and Towbin 2010). It is a frequent cause of heart failure (HF), which leads to heart transplant (HT) at a relatively young age (Felker et al. 2000). Advancement of knowledge in the pathophysiology of HF, efforts to facilitate early diagnosis, and use of effective pharmacological treatments and device therapies have contributed greatly to improving patients’ survival in the last few years. Risk stratification mainly depends on assessment of left ventricular ejection fraction (LVEF) (Gulati et al. 2003). Other commonly known, unfavorable prognostic factors in HF are subdivided into several groups, including demographic, genetic, clinical, and biochemical and neurohormonal activation markers (Braunwald 2008). Among the biochemical parameters, assessment of activity of natriuretic peptide is widely used clinically as a prognostic marker (Lainscak et al. 2009). However, given the complexity of this syndrome, it is unlikely that a single biomarker would improve the risk stratification. In chronic HF, many compensatory mechanisms maintaining perfusion to vital organs become insufficient. The disturbances in local circulation of important organs, and endothelial and neurohormonal dysfunction are associated with oxidant-antioxidant imbalance and consequent subcellular alteration (Bergamini et al. 2009).

Increased total oxidant status and decreased total antioxidant status may indicate an enhanced oxidative stress in patients with HF (Ellidag et al. 2014). Free radicals can be involved in cardiomyocyte hypertrophy, apoptosis, and mechanisms of cardiac remodeling (Nonaka-Sarukawa et al. 2003). Increased myocardial oxidative stress (OS), which is observed in animal models, plays a pivotal role in the pathogenesis of cardiac injury and the progression of HF (Nojiri et al. 2006; Charniot et al. 2011).

Several factors and mechanisms, including xanthine oxidase (XO), NAD(P)H oxidases, cytochrome P450, auto-oxidation of catecholamines, and uncoupling of nitric oxide synthase (NOS) cause an increased production of reactive oxygen species (ROS) in a failing heart (Giordano 2005; Nordberg and Arner 2001).

The primary defense system against oxidative stress consists of antioxidant enzymes, including superoxide dismutase (SOD), catalase, and glutathione peroxidase, heat shock protein enzymes-heme oxygenases (HOs) which have essential antioxidant capacity for many tissues including cardiovascular system cells (Haines and Tosaki 2018) and nonenzymatic components, including glutathione, metallothionein, α-tocopherol, and ascorbate. SOD catalyzes dismutation of O_2_^·−^ to hydrogen peroxide (H_2_O_2_). H_2_O_2_ is a relatively less toxic molecule and under physiological conditions is eliminated by catalase or glutathione peroxidase. However, H_2_O_2_ and O_2_^·−^ may generate highly reactive and toxic hydroxyl radicals (OH^·^). A manganese-containing isoenzyme, manganese superoxide dismutase (MnSOD), located within the mitochondrial matrix, is responsible for prompt conversion of mitochondrial O_2_^·−^ to H_2_O_2_, which is less reactive, and better crosses cell membranes. Copper/zinc-containing SOD (CuZnSOD) is a cytosolic enzyme that catalyzes the same reaction. This enzyme plays a predominant role in superoxide dismutation, although this reaction also occurs nonenzymatically. Our research identified both forms of SOD: the mitochondrial MnSOD and the cytosolic CuZnSOD, along with the extracellular SOD (EC-SOD), which is similar to the cytosolic CuZnSOD (Petersen et al. 2003). MnSOD is of particular interest because of its strategic location in the mitochondria, a major site of O_2_ production under hypoxic conditions (Tsan et al. 1998). Biomarkers including oxidative stress markers may provide valuable new insight into the pathophysiology and prognosis of HF. There is growing evidence that oxidative stress may contribute to disease progression in dilated cardiomyopathy. Hence, the purpose of this study was to critically evaluate superoxide dismutase activity as a predictor of adverse outcomes in patients suffering from heart failure due to dilated cardiomyopathy.

## Methods

### Patients

We recruited 109 consecutive patients aged 49.4 ± 17.5 years with NIDCM, diagnosed according to the World Health Organization criteria (Richardson et al. 1996). All patients were considered to be potential heart transplant recipients and admitted to the Second Department of Cardiology, School of Medicine, with the Division of Dentistry, Medical University of Silesia, Poland, between 2006 and 2011 due to a follow-up procedure. Depending on the results of noninvasive assessment, an invasive assessment (right heart catheterization) was performed in most patients. Patients were clinically stable and their therapy had not been changed for at least 1 month before the enrollment. Ischemic etiology was previously excluded by coronary angiography. All patients in this study were nonsmokers and had not taken any antioxidant supplements and/or vitamins for at least 3 days before the examination. Exclusion criteria included valvular heart disease, significant renal insufficiency, inflammatory or infectious disorders, and other diseases which, by themselves, could have reduced survival. All patients received optimal conventional heart failure therapy, including angiotensin-converting enzyme inhibitors (ACE-I) or angiotensin receptor blockers (ARB), beta-blockers, mineralocorticoid receptor antagonists (MRA), digitalis, and diuretics.

According to the clinical status, the patients were followed on an outpatient basis and examined at least every 6 months, for a period of 5 years. For all patients, endpoint data were collated. The exact date of death was obtained from the patient’s medical history, information from his/her relatives, and, in other cases, from the national identification number database. Written informed consent was obtained from all enrolled patients. The study protocol was approved by the Bioethics Committee of Medical University of Silesia.

### Clinical assessment

Clinical evaluation included a medical examination incorporating assessment of New York Heart Association (NYHA) class, electrocardiogram, and echocardiography. Echocardiography images were acquired in standard views following the recommendations of the American Society of Echocardiography.

### Blood sample collection

Blood samples of all subjects were collected on the day of clinical assessment (which included electrocardiogram and echocardiography at the start of the study). The samples were collected in the morning before breakfast, after 12 h of overnight fasting. All blood samples (5 ml) were taken from the basilic vein into two kinds of tubes: ethylenediaminetetraacetic acid (1.6 mg/ml EDTA-K3) tubes for hemoglobin determination and clot activator containing tubes. The blood samples were centrifuged (10 min, 1500 *g*, 4 °C), and the serum was immediately separated. Routine laboratory determinations were made (creatinine, hemoglobin, sodium, N-terminal prohormone of brain natriuretic peptide [NT-proBNP]) from a portion of the serum collected. The rest of the serum sample was stored at a temperature of − 70 °C, until further biochemical analyses of SOD and malondialdehyde (MDA) were performed.

### Biochemical methods

The serum superoxide dismutase (SOD, EC 1.15.1.1) activity was determined by the Oyanagui method (Oyanagui 1984). In this method, XO catalyzes the oxydation of hypoxanthine to xanthine and generates superoxide in the presence of oxygen. Increasing activity of SOD in the samples causes a decrease in superoxide concentration and a reduction of the product color (naphthyl ethylenediamine and sulfanilic acid).

Activities of SOD isoenzymes, MnSOD and CuZnSOD, were measured with the use of potassium cyanide (KCN) as inhibitor of the CuZnSOD isoenzyme. CuZnSOD activity was taken as the difference between the total SOD activity and the MnSOD activity. The activity of SOD was calculated against a blank probe (containing bi-distilled water). Enzyme activity was expressed as nitrite units (NU) per milliliter serum. One NU exhibits 50% inhibition of formation of nitrite ion under the method’s condition. The inter- and intra-assay coefficients of variations (CV) were 2.8% and 5.4%, respectively.

Malondialdehyde was measured according to the method described by Ohkawa using the reaction with thiobarbituric acid (Ohkawa et al. 1979). NT-proBNP was measured by electrochemiluminescence (Cobas 6000e501, Roche Diagnostics, Mannheim, Germany). Additionally, we also examined hemoglobin, sodium, and creatinine concentrations using routine techniques (Cobas Integra 800, Roche Diagnostics). Glomerular filtration rate (eGFR), an indicator of renal function, was estimated from serum creatinine using the Cockcroft-Gault Equation.

### Endpoints of the study

The endpoints of the study were as follows: heart transplant (HT), all-cause mortality, and combined endpoint (all-cause mortality or heart transplant).

### Statistical analysis

For the purpose of analysis, the study group was divided into four groups according to the outcome: group A, patients who survived without endpoints; group B, patients who underwent a heart transplant; group C, patients who died; and group D, patients who achieved a combined endpoint. Categorical data were presented as numbers and percentages. Because of the small sample size, continuous data were expressed as median and interquartile range (25–75%). The significance of baseline differences between groups was determined using the chi-square test with Yates correction for categorical data or the unpaired Mann-Whitney *U* test for continuous data, as appropriate. The Spearman rank correlation method was used as a nonparametric measure of association for correlations between SOD activities and clinical and biochemical variables. Cumulative survival curves were constructed as time to the endpoint by Kaplan-Meier methods, and the differences between patients, with activities of SOD and its isoenzymes below and above the median, were tested for significance by the log-rank test.

As a first step in the development of risk models, univariate analyses using Cox regression were performed in the study population to determine the relationships between known risk factors and endpoints. The clinical, echocardiographic, and laboratory variables, which differed significantly under comparative statistics, were included in a univariate Cox analysis. Multivariate Cox regression analyses were subsequently performed, with risk factors associated with the combined endpoint in the univariate analysis; only variables with a value of *P* ≤ 0.1 at univariate analysis were included in the multivariate model. Three different models including laboratory parameters and separately, total SOD, MnSOD, and CuZnSOD were fit for use as the combined endpoint. These models were adjusted subsequently for LVEF, body mass index (BMI), and NYHA class. We decided to use LVEF (not the left ventricular end-diastolic volume [LVEDV]), for multivariate analysis. LVEF and LVEDV are derivatives and in the two-factor analysis, a stronger predictive value was demonstrated by LVEF (LVEF: hazard ratio [HR] 0.934, 95% confidence interval [CI] 0.887–0.983; LVEDV: HR 1.028, 95% CI 0.988–1.069). The results of the Cox analysis were reported as relative risks with corresponding 95% CI. The results were considered statistically significant if *P* < 0.05. Statistical analysis was performed with STATISTICA, version 10.0 (StatSoft, Inc., Tulsa, OK, USA).

## Results

### Clinical and laboratory characteristics

A total of 109 patients (16 females; mean age 49.4 [37.6–57.2] years) with NIDCM were examined with a 5-year-long follow-up; of these, 40 (36.7%) patients achieved the endpoint, 15 (13.8%) patients underwent heart transplant, and 25 (22.9%) patients died. All patients received optimal medical treatment according to current guidelines (Swedberg et al. 2005). Majority of them received β-blockers (93.6%), ACE-I (92.7%), ARB (37.6%), and MRA (91.7%). Some patients were treated with digitalis (63.3%), loop diuretics (61.5%), and thiazide diuretics (17.4%). In the presented group, 34 (31.2%) patients were maintained on ACE-I and ARB combination therapy under strict potassium and creatinine control.

Thirty-three percent of the patients had an implantable cardioverter-defibrillator. Patients from groups C and D received loop diuretics more frequently. Otherwise, pharmaco- and electro-therapies were not different. The demographic, clinical, and echocardiographic baseline characteristics for all groups and subgroups are presented in Table 1, and laboratory parameters are shown in Table 2.

**Table 1:** Demographic, clinical, and selected echocardiography date in subgroups separated according to prognosis

	A	B	C	D
	Survivors	HT	Death	HT or death
	*n* = 69	*n* = 15	*n* = 25	*n* = 40
Sex (female), *n* (%)	10	2	4	6
	(14.5)	(13.3)	(16)	(15.0)
Age (years)	49.39	49.06	50.30	49.44
	(37.56–56.51)	(42.99–54.76)	(36.45–87.90)	(38.58–57.90)
BMI (kg/m^2^)	28.37	25.53^AA^	27.68	25.83^AA^
	(25.78–31.35)	(21.81–26.65)	(23.77–29.70)	(22.64–29.05)
NYHA class I–II/III–IV (%)	51/18	4/11^AAA^	15/10	19/21^AA^
	(73.9/26.1)	(26.6/73.4)	(60.0/40.0)	(47.5/52.5)
Disease duration (years)	3.21	5.59^A^	3.19	4.36
	(1.38–6.15)	(4.05–9.36)	(0.76–5.51)	(1.71–6.15)
ICD, *n* (%)	23	7	3	10
	(20.9)	(46.7)	(12.0)	(25.0)
HA, *n* (%)	20	2	7	9
	(29.0)	(13.3)	(28.0)	(22.5)
DM t. 2, *n* (%)	8	1	2	3
	(11.6)	(6.7)	(8.0)	(7.5)
Sinus rhythm, *n* (%)	55	12	21	33
	(79.7)	(80.0)	(84.0)	(82.5)
Atrial fibrillation, *n* (%)	14	3	4	7
	(20.3)	(20.0)	(16.0)	(17.5)
LVEF (%)	25.00	20.00^AA^	20.00^A^	20.00^AAA^
	(20.00–30.00)	(15.00–21.00)	(15.00–26.00)	(15.00–25.00)
LVEDV (ml)	185.0	219.0	238.0^A^	229.0^A^
	(153.0–240.0)	(181.5–265.0)	170.0–297.0)	(178.0–270.0)
LVEDD (mm)	68.00	73.00^A^	69.00	71.00
	(62.00–74.00)	(68.00–78.50)	(62.00–80.00)	(65.00–80.00)

*HT* heart transplant, *BMI* body mass index, *NYHA class* New York Heart Association functional class, *ICD* implantable cardioverter-defibrylator, *HA* arterial hypertension, *DM t.2* diabetes t. 2, *LVEF* left ventricle ejection fraction, *LVEDV* left ventricle end-diastolic volume, *LVEDD* left ventricle end-diastolic diameter

Statistic significance: ^A^*p* < 0.05, ^AA^*p* < 0.01, ^AAA^*p* < 0.001 when compared to A group

**Table 2:** Activity of serum biomarkers in subgroups separated according to prognosis

	A	B	C	D
	Survivors	HT	Death	HT or death
	*n* = 69	*n* = 15	*n* = 25	*n* = 40
NTproBNP	605.0	2395^AAA^	1420^AA,B^	1606^AAA^
[pg/ml]	281.0–1270	1135–3000	832.0–1903	876.0–3000
CC	120.0	91.40^AA^	100.6	99.05^AA^
[ml/min]	100.6–149.8	74.15–112.2	92.31–138.6	83.87–134.7
Sodium	138.0	135.0^A^	138.0	135.5^A^
[mmol/l]	136.0140.0	124.0–137.0	133.0–140.0	131.0–139.5
SOD	10.48	17.49^AA^	18.23	17.70^AA^
[NU/ml]	7.46–18.18	16.64–22.22	8.55–21.80	13.79–22.07
MnSOD	7.25	10.96	12.05^A^	11.23^AA^
[NU/ml]	3.37–12.89	9.138–12.12	5.10–15.30	5.77–15.06
CuZnSOD	4.92	6.52^A^	4.83	5.28
[NU/ml]	2.37–6.53	4.52–8.64	3.71–7.33	4.20–7.58
MDA	4.51	4.37	4.22	4.26
[μmol/l]	3.89–5.90	3.40–5.67	3.51–4.87	3.40–4.96
Hemoglobin	144.0	131.5^A^	141.0	138.0^A^
[g/dl]	136.0–154.0	126.0–142.0	130.0–153.0	128.0–151.0

*HT* heart transplant, *NT-proBNP* N**-**terminal pro-B-type natriuretic peptide, *CC* creatinine clearance, *SOD* superoxide dismutase, *MnSOD* manganese superoxide dismutase, *CuZnSOD* copper-zinc superoxide dismutase, *MDA* malondialdehyde

Statistic significance: ^A^*p* < 0.05, ^AA^*p* < 0.01, ^AAA^*p* < 0.001 when compared to A group; ^B^*p* < 0.05 when compared to B group

The percentage of comorbidities was low, and it did not differ between groups. More patients from groups B and D were in the advanced NYHA functional class (III and IV) as compared to group A. Echocardiography showed a typical enlargement of the left ventricle and markedly depressed LVEF (25.0 [20.0–30.0%]). LVEF values in patients who achieved endpoints (groups B, C, and D) were significantly lower as compared to group A. Groups B, C, and D had significantly higher NT-proBNP concentration and lower creatinine clearance compared to group A. The hemoglobin and sodium concentrations were lower in groups B and D.

### Comparison of activities of total SOD and its isoenzymes in patients with dilated cardiomyopathy group according to the prognosis

Total SOD activity was statistically significantly higher in the HT and combined endpoint groups as compared to patients who survived without endpoint (*p* < 0.01). MnSOD activity was statistically significantly higher in patients who died or achieved the combined endpoint (*p* < 0.05, *p* < 0.01), whereas CuZnSOD activity was statistically significantly higher only in HT patients as compared to patients who survived without endpoint during the 5-year follow-up (*p* < 0.05).

### Correlation between SOD isoenzymes and clinical and biochemical parameters

We observed significant correlation between SOD isoenzymes and severity of HF (as defined by LVEF, NYHA class, and NT-proBNP) in all the groups. Furthermore, the total SOD and MnSOD activities correlated negatively with BMI and creatinine clearance (Table 3). Surprisingly, there was no correlation between SOD and MDA.

**Table 3:** The Spearman’s Rank correlation between SOD izoenzymes and clinical and biochemical parameters

	Total SOD		MnSDOD		CuZnSOD	
	*R*	*P*	*R*	*P*	*R*	*P*
Age	− 0.061	NS	− 0.023	NS	− 0.095	NS
BMI	− 0.250	0.010	− 0.253	0.009	− 0.145	NS
NYHA class	0.218	0.023	0.175	NS	0.156	NS
Disease duration	0.017	NS	− 0.002	NS	− 0.028	NS
LVEF	− 0.317	0.001	− 0.270	0.004	− 0.211	0.028
LVEDV	0.140	NS	0.182	0.058	− 0.026	NS
LVEDD	0.026	NS	0.049	NS	− 0.065	NS
NTproBNP	0.297	0.002	0.260	0.008	0.217	0.028
CC	− 0.211	0.029	− 0.202	0.037	− 0.128	NS
Sodium	0.009	NS	− 0.005	NS	0.071	NS
MD	0.052	NS	0.088	NS	− 0.078	NS
Hemoglobin	0.063	NS	0.042	NS	0.041	NS

*BMI* body mass index, *NYHA class* New York Heart Association functional class, *LVEF* left ventricle ejection fraction, *LVEDV* left ventricle end-diastolic volume, *LVEDD* left ventricle end-diastolic diameter, *NT-proBNP* N-terminal pro-B-type natriuretic peptide, *CC* creatinine clearance, *MDA* malondialdehyde, *NS* statistically non-significant results, *R* Spearman’s Rank Correlation Coefficient, *P* statistical significance

### Follow-up and predictors of unfavorable outcomes

**Fig. 1:**
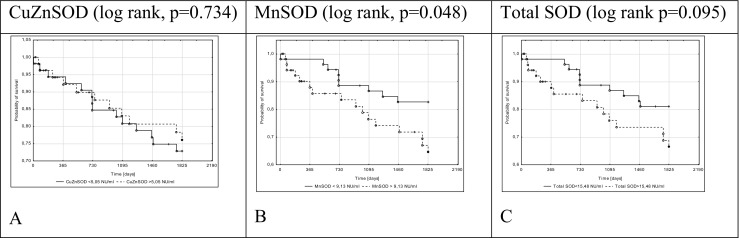
Kaplan–Meier death-free survival in 5-year long follow-up depending on total SOD (**c**), MnSOD (**b**), and CuZnSOD (**a**) activity in NIDCM patients

**Fig. 2:**
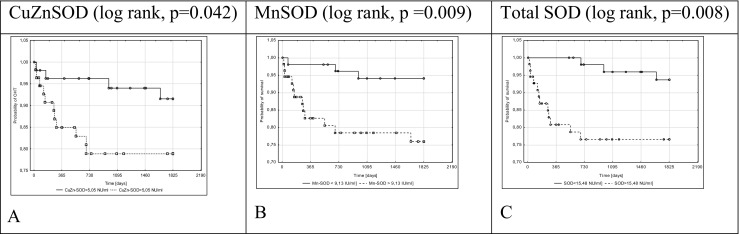
Kaplan–Meier heart transplantation-free survival in 5-year long follow-up depending on total SOD (**c**), MnSOD (**b**), and CuZnSOD (**a**) activity in NIDCM patients

**Fig. 3:**
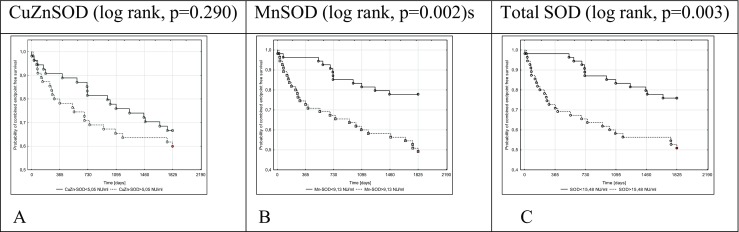
Kaplan–Meier death- or heart transplantation-free survival in 5-year long follow-up depending on total SOD (**c**), MnSOD (**b**), and CuZnSOD (**a**) activity in NIDCM patients

To evaluate the efficacy of SOD activity as a prognostic factor for adverse clinical outcome in NIDCM, we subdivided the patients according to their SOD activity into two groups: patients with SOD activity below and those with SOD activity above the median SOD activity. Kaplan-Meier curves based on SOD, MnSOD, and CuZnSOD activities for all-cause mortality, heart transplant, and combined endpoint groups are shown in Figs. 1, 2, and 3, respectively.

Log-rank analysis for all endpoints revealed that a serum MnSOD activity > 9.13 NU/ml was a predictor of poor prognosis in patients with NIDCM (Figs. 1B, 2B, and 3B). Stratification based on the median SOD activity revealed that patients with higher SOD (total SOD activity > 15.48 NU/ml) underwent HT or achieved the combined endpoint more often. CuZnSOD was useful only in the prediction of HT in patients with NIDCM.

**Fig. 4:**
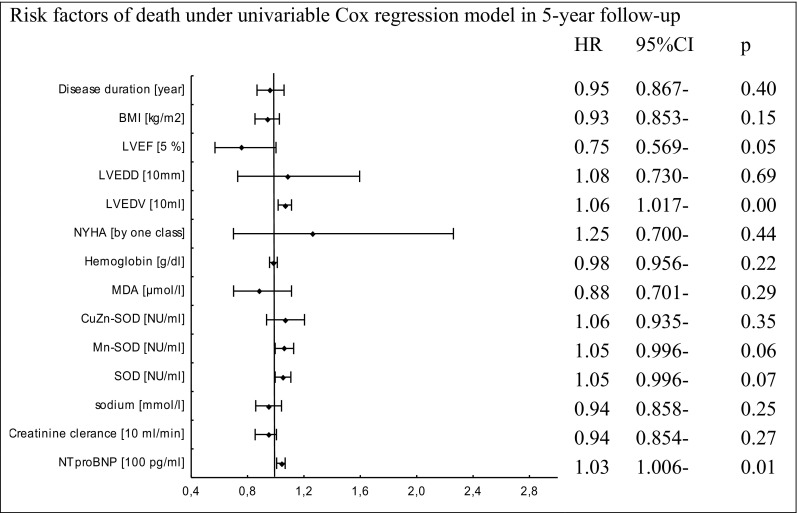
Cox proportional-hazards regression for 5-year mortality rate in relation to biochemical, demographical, and clinical factors

**Fig. 5:**
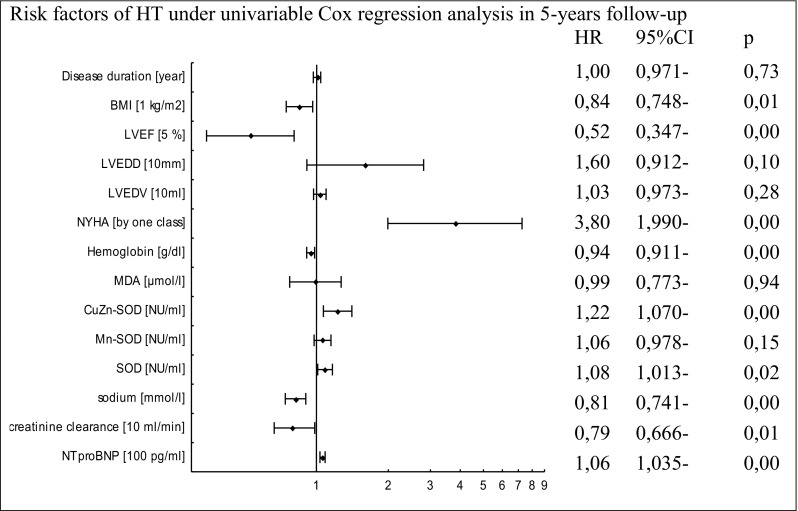
Cox proportional-hazards regression for HT rate in relation to biochemical, demographical, and clinical factors

**Fig. 6:**
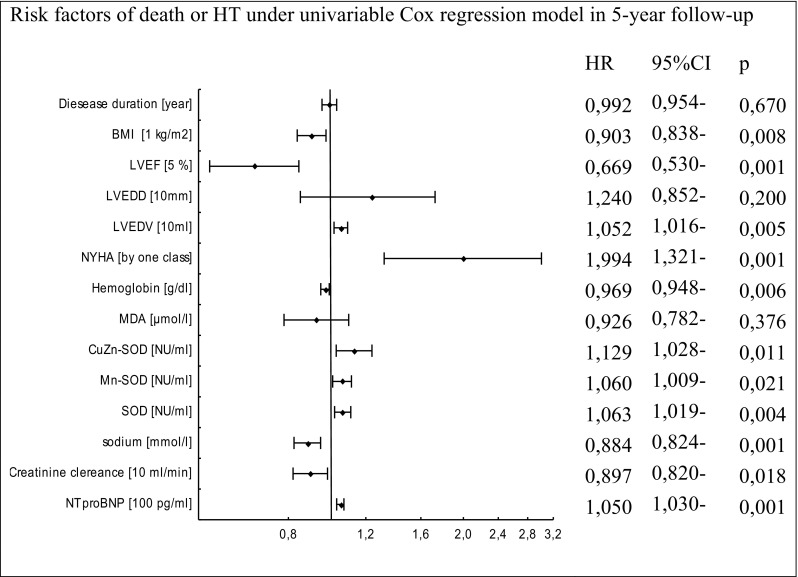
Cox proportional-hazards regression for 5-year mortality or HT rate in relation to biochemical, demographical, and clinical factors

Univariate analysis identified only higher concentration of NT-proBNP and increased LVEDV as predictors of death (Fig. 4). In univariate analysis, poor prognostic factors for HT in this study group were lower levels of the following parameters: BMI, LVEF, hemoglobin, sodium, and higher levels of the following indices: NYHA class, NT-proBNP concentrations, creatinine clearance, SOD, and CuZnSOD activities (Fig. 5). Similarly, the above factors and higher MnSOD activity and LVEDV were associated with combined endpoint occurrence (Fig. 6). Multivariate analysis was performed for the combined endpoint only. This was dictated by a limited number of completed endpoints.

**Fig. 7:**
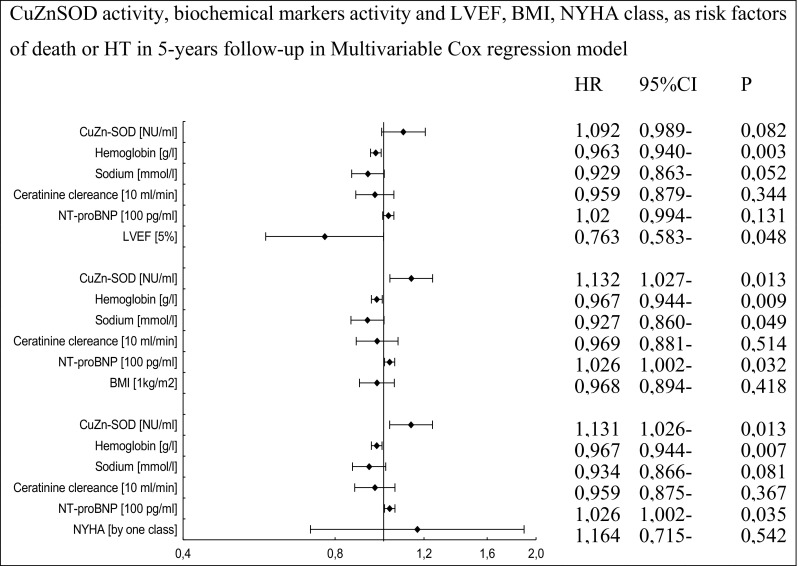
Multivariable Cox proportional-hazards regression for CuZnSOD activity, LVEF, BMI, NYHA class, and biochemical parameters as risk factors of death or HT in 5-year follow-up

**Fig. 8:**
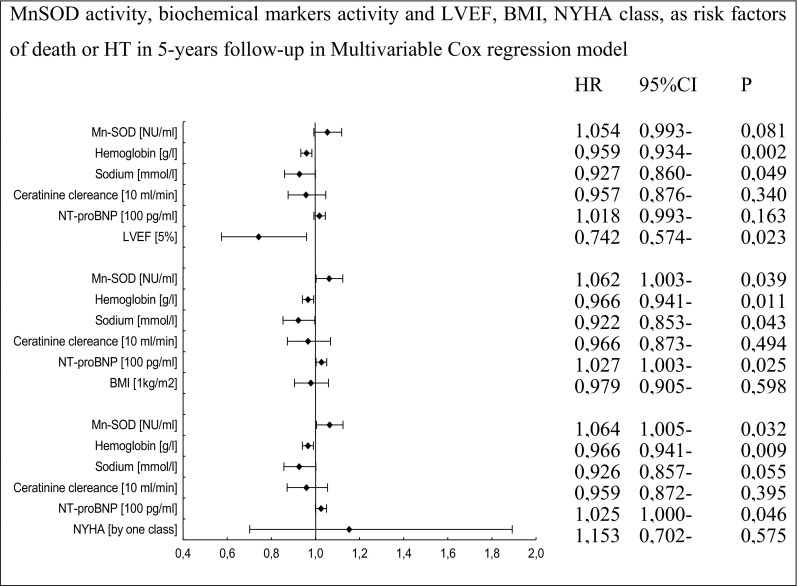
Multivariable Cox proportional-hazards regression for MnSOD activity, LVEF, BMI, NYHA class, and biochemical parameters as risk factors of death or HT in 5-year follow-up

In multivariate analysis, activities of total SOD and its isoenzymes, MnSOD and CuZnSOD, remained independent prognostic factors of the combined endpoint even after adjustment for all significant biochemical predictors, BMI, and NYHA class.

**Fig. 9:**
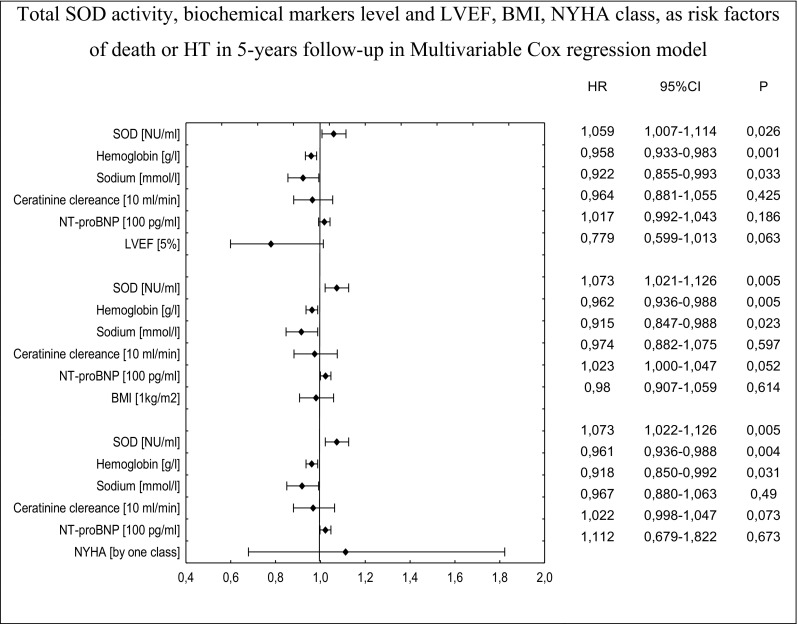
Multivariable Cox proportional-hazards regression for total SOD activity, LVEF, BMI, NYHA class, and biochemical parameters as risk factors of death or HT in 5-year follow-up

The inclusion of LVEF in the multivariate model did not abolish the prognostic significance of activity of total SOD. In this regression model (taking into account the LVEF value), activities of CuZnSOD and MnSOD, and the concentration of NT-proBNP did not reach statistical significance (Figs. 7, 8, and 9).

## Discussion

This study is the first to assess the impact of SOD activity on the long-term prognosis of patients with nonishemic dilated cardiomyopathy. Increased activities of total SOD and its isoenzymes predicted death and/or the necessity of a heart transplant during the 5-year follow-up period. Oxidative stress and free radicals are described as the main causes of nonischemic cardiomyopathy in experimental and clinical studies. For example, enhanced generation of ROS seems to be crucial in explaining the toxicity of ethanol in alcoholic cardiomyopathy (Eleawa et al. 2015). The contribution of oxidative stress is also well established in diabetic cardiomyopathy (Steiner and Lang 2017) and endemic cardiomyopathy in Keshan disease (Pei et al. 2013) or Chagas disease (Machado et al. 2013). The heart is particularly susceptible to oxidative damage, as it possesses lower levels of free radical scavengers such as CuZnSOD, catalase, and glutathione peroxidase, as compared to the liver (Chen et al. 1994). A profound impact on mitochondrial superoxide dismutase (MnSOD) has been shown in anthracycline-induced cardiomyopathy. Systemic antracycline treatment markedly downregulates the expression of MnSOD both at the level of mRNA and protein and decreases its enzymatic activity for up to 3 weeks. (Li et al. 2000). Though the abnormalities in some cardiomyopathies have been well identified, comprehensive understanding of underlying mechanisms of the myocardial remodeling process resulting in the progression of heart failure is needed. Hence, we decided to assess the usefulness of SOD in a group of patients with nonischemic dilated cardiomyopathy. The study group consisted of clinically stable patients. The proportion of patients with additional illnesses such as diabetes or hypertension was low (no complications of hypertension and diabetes were noted). Patients were young and had significantly reduced LVEF. Though endomyocardial biopsy is a well-known, sensitive, and reliable detector of oxidative stress in cardiac tissue, its invasive nature (Pei et al. 2013) significantly limits its routine use in clinical practice. Thus, we analyzed SOD activity not in heart tissue but from blood, so such factors as peripheral congestion and hypoxic condition may influence the results. However, careful patient selection ensured that we could evaluate redox parameters in HF regardless of the impact of the endothelial processes on redox balance occurring in atherosclerosis or advanced arterial hypertension. Previous studies indicate that serum SOD activity and MDA are higher in patients with NIDCM; only isoenzyme MnSOD is related to severity of HF (Wojciechowska et al. 2014). Similar results showing an inverse correlation of SOD activity with the cardiac index were documented by Jacheć et al. (1998). Association between SOD and glutathione reductase (GR) and severity of LV dysfunction (higher SOD and GR associated with lower LVEF, not at rest but after cardiopulmonary exercise testing) in NIDCM were observed by Simeunovic et al. (2015).

We have observed an association between the severity of LV dysfunction and SOD and GR. i.e., higher SOD and GR were associated with lower LVEF; this was observed after cardiopulmonary exercise testing in NIDCM. Keith et al. (1998) found no correlations between lipid peroxidation, MDA, and ejection fraction or LV function or dimensions (as determined by echocardiography). However, these parameters were positively correlated with the functional class of HF. Thus, we assessed the long-term prognostic and predictive value of serum SOD activity in NIDCM, together with known prognostic parameters, such as BMI, NYHA class, and LVEF. SOD had a long-term prognostic-predictive value even after adjustment for previously established prognostic factors, such as NT-proBNP, sodium and hemoglobin concentrations, creatinine clearance, LVEF, NYHA class, or BMI. The predictive efficacy of MnSOD and CuZnSOD for the combined endpoint was independent of laboratory parameters, NYHA class, and BMI. The clinical value of serum biomarkers in prognostic stratification lies in their ability to identify patients with an unfavorable prognosis more accurately, thus facilitating intensification of treatment in this subset of patients. The impact of superoxide dismutase-1 genetic variation on all-cause mortality was evaluated in a prospective cohort study by Otaki et al. (2016). Haplotype analysis revealed that some haplotypes increased, whereas others decreased the likelihood of death due to cardiovascular diseases. However, protein expression and activity of SOD1 were not measured. Only one previous study has established the prognostic role of serum SOD in cardiovascular disease (Parenica et al. 2016). SOD was useful as a predictor of 1-year all-cause mortality and/or hospitalization due to acute HF in patients after ST-elevation myocardial infraction. In the above study, patients had more frequent comorbidities and only mild postinfarct dysfunction of the left ventricle. These conditions and atherosclerotic coronary arterial lesions may affect the degree of disturbances in the redox balance parameters in this group of patients. However, SOD was an independently powerful prognostic factor in addition to the GRACE score. SOD also demonstrated a significant ability to predict endpoints in 2-year and 3-year follow-ups (Parenica et al. 2016). The association between serum oxidative stress markers and functional class (Wojciechowska et al. 2014; Keith et al. 1998) and the results after effort (Simeunovic et al. 2015) may support the concept that redox markers are related not only to cardiac contractility and cardiac output but also to impairment of peripheral blood flow, hypoxia, or skeletal muscle function. Surprisingly, multivariate analysis in our study abrogated the prognostic value of NYHA functional class of HF. Experimental studies have demonstrated the biological importance of SOD. In mice lacking the following mitochondrial SOD mutant, severe effects, such as inactivation of key respiratory chain and tricarboxylic acid cycle enzymes, inhibition of HMG-CoA lyase, and accumulation of oxidative DNA damage were seen (Meelov et al. 1999).

In the context of HF, most free radicals are thought to emanate from inefficient mitochondrial metabolism and/or impaired antioxidant defense (Seddon et al. 2007). MnSOD is the only SOD isoform that is essential for the survival of aerobic organisms (Carlioz and Touati 1986; Lebovitz et al. 1996). Located in the mitochondria, MnSOD makes up > 70% of the SOD activity in the heart and > 90% of the activity in cardiac myocytes. The remaining activity of SOD is due to CuZnSOD, which is located in the cytosol (Sawyer et al. 2002). Our results demonstrate a key prognostic role of MnSOD. Log-rank analysis demonstrated that high serum MnSOD activity was a significant predictor of death, HT, or combined endpoint. Similar to Dieterich et al. (2000), we conclude that the increase in antioxidant enzyme expression is evidence of an adaptive response to the increased substrate concentration in mild oxidative stress. In an early animal study, Gupta and Singal (1989) found increased antioxidant activity consistent with the notion that oxidative stress is associated with increase in antioxidant enzyme activity. In a study by Sam et al. (2006), only CuZnSOD activity was increased in HF. Despite increase in the general expression of MnSOD, the activity of this enzyme was decreased. Taking into consideration different biochemical markers, the NT-proBNP is a new marker, which is very strongly associated with adverse outcome in patients with HF. Brain natriuretic peptide is commonly used as a diagnostic marker for HF (McDonagh et al. 2004). A previous study reports that BNP is also a predictor of all-cause and cardiovascular mortality in the general population (Parenica et al. 2016). It is worth emphasizing that carriers of superoxide dismutase-1 genetic variation associated with increased cardiovascular mortality have elevated BNP levels (Otaki et al. 2016). Surprisingly, in our study, the predictive significance of NT-proBNP was attenuated when LVEF was included in the multivariate model. Another marker of poor prognosis in HF is low hemoglobin concentration. Under hypoxic conditions, as in heart failure, hemoglobin can contribute to production of ROS because heme releases superoxide during desaturation (Tang and Katz 2006). In patients with dilated cardiomyopathy, lower hemoglobin concentration was a useful predictor of heart transplant and combined endpoint. The association between anemia and SOD activity shows inconsistent results. Jansson et al. (1985) observed increased formation of SOD in iron-deficient rats during increased oxidative stress*.* Cellerino et al. (1976) reported enzyme deficiency in severe iron-deficiency anemia. We did not observe any difference in the MDA concentration between groups of patients with adverse outcomes and those who survived. We did not show that MDA from lipid peroxidation can predict mortality. Inverse correlations between the concentration of MDA and mixed venous blood oxygenation and also with total antioxidant capacity were previously observed in patients with dilated cardiomyopathy (Wojciechowska et al. 2014). This perhaps suggests that existing antioxidant defense mechanisms in stable patients are sufficient to inactivate the production of ROS. In summary, the present study demonstrates that the activities of SOD and its isoenzymes are useful predictors of adverse outcome in patients with reduced ejection fraction heart failure due to dilated cardiomyopathy. Total SOD, MnSOD, and CuZnSOD offer a moderate improvement in risk stratification when used in combination with conventional markers and improve the assessment of prognosis beyond established clinical risk scores. However, our results need further validation and confirmation in future cohort studies. The next logical step is to obtain a nomogram by measuring representatives of distinct classes of antioxidant and oxidant factors. Furthermore, antioxidant activity has a therapeutic potential, which could reduce morbidity and mortality in patients with dilated cardiomyopathy.

### Study limitations

We do, however, acknowledge that our study has certain limitations. First, the study group was relatively small. However, the group was homogenous, of non-ischemic etiology, and patients with exacerbation of HF were not included in the study. Second, when there are a small number of endpoints, especially a combined endpoint in relation to variables in a multivariate analysis, further observation is necessary; this was not available in our study. Third, we analyzed the concentrations of biomarkers only at recruitment. They were not re-evaluated during follow-up. Finally, during follow-up, therapy was modified according to the clinical status. Thus, treatment effect during follow-up cannot be ruled out.

## Data Availability

All of the data used to support the findings of this study are included within the article.
